# Supplementary material for: A win-win scenario? Employers’ responses to HIV in Tanzania: A qualitative study
Source: PLOS Glob Public Health. 2022 Nov 21;2(11):e0000058. doi: 10.1371/journal.pgph.0000058 (PMC10021273; doi:10.1371/journal.pgph.0000058)
Supplement: S1 File — (DOCX) [file pgph.0000058.s002.docx]

**IDI Researchers Guide: Businessmen and attitudes towards HIV Testing**

Introductions

Discussing the information sheet and consent form

Why do we want to do the study?

- There are still significant numbers of men in the wealthiest quintile in Tanzania who have not taken a HIV test
- Businessmen have not been included in any HIV research before
- Getting tested is of vital importance to help men access treatment

What are the potential benefits?

The information you give us will:

- Help us design policies and programmes that will help us to encourage more businessmen to test for HIV
- Help us to design policies and programmes that will help us to encourage more men to test for HIV
- Help us understand the impact of the HIV epidemic on businesses

Why have we invited you to participate?

- You are an owner/director/manager/HR manager of a large business in Mwanza that employs a lot of people

Do not switch on the tape recorder until the participant has read and signed the information sheet and consent form and has had a chance to ask any questions.

**A) Demographic information**

- Age
- Marital status
- Brief details of business/organisation/company
  - Sector
  - Main activities
  - Number of employees/ Size
  - When the business/company/organisation was established?

**B) HIV and your business**

- What do you think about HIV?
- Has HIV ever been an issue that has had an impact on your business/organisation/company?
  - If so, in what ways?
  - What (if anything) have you done about it?
- Do you provide HIV testing for your employees?
  - Why yes? Or Why No? Where?
  - If yes, what are the views of your employees about this?
- Do you support your employees to help them access ARV’s and treatment? In what ways?
- Do you have a HIV policy in your business/organisation?
  - If yes, could you please tell us about it?
    - What were the reasons that made you have one?
  - If no, could you explain a bit more about why not?
    - Is this something that your organisation has ever considered?
  - Do you have any programmes on HIV at your business/organisation?
    - If yes, please could you tell us a bit more about it?
    - If no, have you ever considered having one?
  - If yes, Why? If no, Why not?

**C) Attitudes towards HIV testing (general)**

- What do people in this community think about HIV testing?
- What types of people usually test for HIV? Please explain?
- Do you think that other people in similar positions to yours would test for HIV?
  - If yes, why do they?
  - If no, why may some people in similar positions to yours not take a HIV test?
    - What are the main reasons?
  - Do you think that people in a similar position to yours get infected with HIV?
    - If yes, could you tell us more about why they might?
    - If no, why do you think so?
  - How about people’s wealth status? Do you think it plays a role?

(note – we are interested in their views about whether HIV is related to poverty)

**D) Attitudes towards HIV testing (personal)**

Note - Cover this section only if they have not discussed this earlier

Link “We have talked about some of these things in general, now we would like to ask you a few personal questions about your own experience on these things, would that be okay? Thank you”

- Have you ever taken a HIV test?
  - If yes, what are some of the reasons for taking the test?
    - Were there any issues that made you consider not taking a test?
    - What was your experience taking the test?
      - Could you tell us about some of the difficulties?
      - How did you go about it?
      - Were there things that made it easy for you to test?
  - If no, what were the reasons?
- What you think should be done to enhance HIV testing in people in a similar position to yours?

**End of interview**

- Thank you very much for participating in this discussion
- Is there anything else they think we should know about this topic that we have not covered today?
- Do you have any questions?
- Thank you again
