# Supplementary material for: A win-win scenario? Employers’ responses to HIV in Tanzania: A qualitative study
Source: PLOS Glob Public Health. 2022 Nov 21;2(11):e0000058. doi: 10.1371/journal.pgph.0000058 (PMC10021273; doi:10.1371/journal.pgph.0000058)
Supplement: S2 File — (DOC) [file pgph.0000058.s003.doc]

**Muongozo wa Mtafiti wa Mahojiano ya Kina: Wanaume wenye kipato kikubwa na mtazamo juu ya upimaji wa VVU**

**Utangulizi**

Kujadili taarifa na fomu ya ridhaa

**Kwa nini tunataka kufanya utafiti?**

- Bado kuna idadi kubwa ya wanaume wenye kipato kikubwa nchini Tanzania ambao hawajapima VVU
- Wanaume wenye kipato kikubwa hawajajumuishwa katika tafiti yeyote ya VVU kabla
- Kupima ni kitu muhimu kwa kusaidia wanaume kufikia matibabu

**Je, kuna faida gani?**

Taarifa utakazotupati zitaweza:

- Kutusaidia kuandaa sera na miradi itakayotusaidia kuhamasisha wanaume wenye kipato kikubwa wengi zaidi kupima VVU
- Kutusaidia kuandaa sera na miradi itakayotusaidia kuhamasisha wanaume wengi zaidi kupima VVU
- Kutusaidia kuelewa matokeo ya janga la VVU kwenye biashara

**Kwa nini tumekuarika wewe kushiriki?**

- Wewe ni mmiliki/mkurugenzi/meneja/meneja Rasimali Watu wa biashra kubwa mkoani Mwanza inayoajiri watu wengi

Usiwashe kinasa sauti mpaka mshiriki asome na asaini taarifa na fomu ya ridhaa na amepata nafasi ya kuuliza swali lolote.

**A) Taarifa za binafsi**

- Umri
- Hali ya ndoa
- Taarifa fupi za biashara/shirika/kampuni
- Sekta
- Shughuli Kuu
- Idadi ya waajiriwa/ Ukubwa
- Ni lini biashara/kampuni/shirika lilianzishwa?

**B) VVU na biashara yako**

- Unafikiria nini kuhusu VVU?
- Je, VVU vimehi kuwa tatizo ambalo lina madhara katika biashara/shirika/kampuni yako?
- Kama hivyo, kwa namna gani?
- Umefanya nini (kama kuna chochote kilichofanyika) kuhusu hilo?
- Je, unawapima VVU watumishi wako?
- Kwa nini ndiyo? Au Kwa nini Hapana? Wapi?
- Kama ndiyo, yapi maoni ya watumishi wako kuhusu hili?
- Je, unawasaidia watumishi wako kufikia dawa ya kufubaza VVU (ARV's) na matibabu? Kwa namna gani?
- Je, una sera ya VVU katika biashara/shirika lako?
- Kama ndiyo, unaweza kutuambia kuhusu hilo tafadhali?
- Ni sababu gani zilikufanya uwe nayo?
- Kama hapana, unaweza kuelezea kidogo kuhusu kwa nini huna?
- Je, hiki ni kitu ambacho shirika lako limewahi kukitilia maanani?
- Je, una miradi yeyote ya VVU katika bashara/shirika lako?
- Kama ndiyo, unaweza kutuambia zaidi kuhusu hilo tafadhali?
- Kama hapana, je, umewahi kutilia maanani kuwa nayo?
- Kama ndiyo, Kwa nini? Kama hapana, Kwa nini hamna?

**C) Mtazamo juu ya upimani wa VVU (kwa ujumla)**

- Watu katika jamii hii wanafikiri nini kuhusu upimaji wa VVU?
- Ni watu gani hasa mara nyingi hupima VVU? Tafadhali elezea?
- Je, unafikiri kwamba watu wengine walipo kwenye nafasi kama yako wangepima VVU?
- Kama ndiyo, kwa wangepima?
- Kama hapana, kwa nini baadhi ya watu waliopo kwenye nafasi kama yako wasingepima VVU?
- Ni sababu gani kuu?
- Je, unafikiri kwamba watu wengine walipo kwenye nafasi kama yako wanapata maambukizi ya VVU?
- Kama ndiyo, unaweza kutuambia zaidi kwa nini wanaweza kuambukizwa?
- Kama hapana, kwa nini unafikiri hivyo?
- Vipi kuhusu watu wenye kipato kikubwa? Je, unadhani inachangia?

(Kumbuka– Tunavutiwa kwenye maoni yao kuhusu kama VVU vinahusiana na umasikini)

**D) Mtazamo juu ya upimaji wa VVU (binafsi)**

Kumbuka - Uliza kipengele kama tu hakukijadilia mwanzoni

Kiunganishi “Tumeongelea kuhusu vitu hivi kwa ujumla, sasa tungependa kukuuliza maswali machache binafsi kuhusu uzoefu wako mwenyewe katika vitu hivi, je hii ni sawa? Asante"

- Je, umewahi kupima VVU?
- Kama ndiyo, ni sababu gani zilizokufanya upime?
- Je, kulikuwa na masuala yoyote yaliyokufanya usiwaze kupima?
- Uzoefu wako ni upi wa kupima?
- Je, unaweza kutuambia baadhi ya ugumu?
- Je, ulifanyaje kukabiliana na ugumu huo?
- Je, kulikuwa na vitu ambavyo vilifanya iwe rahi kwako kupima?
- Kama hapana, sababu zilikuwa ni zipi?
- Je, unafikiri nini kingefanyika akuimarisha upimaji wa VVU kwa watu waliopo kwenye nafasi kama yako?

**Mwisho wa mahojiano**

- Asante sana kwa kushiriki katika majadiliano haya
- Je, kuna kitu kingine chochote ambacho unafikiri tungekifahamu kuhusu maada hii ambacho hatujakigusia leo?
- Je, una swali lolote?
- Asante sana kwa mara nyingine tena.
