# Supplementary material for: A win-win scenario? Employers’ responses to HIV in Tanzania: A qualitative study
Source: PLOS Glob Public Health. 2022 Nov 21;2(11):e0000058. doi: 10.1371/journal.pgph.0000058 (PMC10021273; doi:10.1371/journal.pgph.0000058)
Supplement: Attachment — Submitted filename: Response to Reviewers comments FINAL.docx [file pgph.0000058.s004.docx]

Additional Editor Comments (if provided):

Specific to the methods section, I highly recommend a more structure approach to the section to include headings for study design (as one is not stated in the manuscript), setting and participants (including the sampling technique), data collection (recording and transcription were not detailed), ethical approval (specific protocol numbers not provided), data analysis (process for analysis was not described), and trustworthiness (elements were not discussed). In addition, the introduction requires the last paragraph to provide the purpose of the research rather than a discussion about the findings of the study. Furthermore, the discussion section requires additional information about the global context of the work in relationship to the findings of the current study. Also, the conclusion seems to include more discussion rather than a summary of the key points to take away from the study. Finally, the reference need to be updated as 22 of the 30 were published prior to 2015, with more than half of these prior to 2010.

Author’s response:

The methods section has been significantly revised, expanded to ensure that it is clearly structured in line with the Editors suggestions and also those of the reviewers.

The introduction, discussion and conclusion have also been edited in line with the editors comments.

The references have been added to and updated, though this a slightly older body of work that needs updating (hence this submission).

Reviewers' comments:

Reviewer's Responses to Questions

**Comments to the Author**

1. Does this manuscript meet PLOS Global Public Health’s [publication criteria](https://journals.plos.org/globalpublichealth/s/criteria-for-publication)? Is the manuscript technically sound, and do the data support the conclusions? The manuscript must describe methodologically and ethically rigorous research with conclusions that are appropriately drawn based on the data presented.

Reviewer #1: No

Reviewer #2: Partly

2. Has the statistical analysis been performed appropriately and rigorously?

Reviewer #1: Yes

Reviewer #2: N/A

3. Have the authors made all data underlying the findings in their manuscript fully available (please refer to the Data Availability Statement at the start of the manuscript PDF file)?

The [PLOS Data policy](https://journals.plos.org/globalpublichealth/s/data-availability) requires authors to make all data underlying the findings described in their manuscript fully available without restriction, with rare exception. The data should be provided as part of the manuscript or its supporting information, or deposited to a public repository. For example, in addition to summary statistics, the data points behind means, medians and variance measures should be available. If there are restrictions on publicly sharing data—e.g. participant privacy or use of data from a third party—those must be specified.

Reviewer #1: No

Reviewer #2: No

4. Is the manuscript presented in an intelligible fashion and written in standard English?

PLOS Global Public Health does not copyedit accepted manuscripts, so the language in submitted articles must be clear, correct, and unambiguous. Any typographical or grammatical errors should be corrected at revision, so please note any specific errors here.

Reviewer #1: No

Reviewer #2: Yes

5. Review Comments to the Author

Please use the space provided to explain your answers to the questions above. You may also include additional comments for the author, including concerns about dual publication, research ethics, or publication ethics. (Please upload your review as an attachment if it exceeds 20,000 characters)

Reviewer #1: Reviewer's report on the article titled “A win-win scenario? Employers’ responses to HIV in sub-Saharan Africa: qualitative evidence from Tanzania”

Date: 4th August 2021

General Comment: The authors are addressing an important subject particularly within the context of a developing country like Tanzania where about 4.9% of the active population (aged 15 years and above) live with HIV and that, discriminatory attitudes towards people living with HIV are still prevalent. However, the manuscript has one major limitation related to the design of the study as detailed in paragraph 1.


1. Sub-Saharan African (SSA) region has more than 40 countries. Accordingly, the findings and associated conclusions and policy implications presented in this manuscript do not offer an opportunity to understand how HIV is addressed in workplaces in SSA. Institutional or workplace responses to HIV are largely dependent on national policies and strategies on HIV prevention and treatment that are in place, which are likely to differ from one country to another. Evidence from a multi-country study design would have added value to the present study. For example, the authors state in lines 19 and 20 that “Our findings suggest that HIV is not generally a big issue, and hence HIV interventions are primarily ad-hoc with few formal HIV workplace programmes”. This is not likely to be case in other countries in SSA. Indeed, the authors point out in lines 75 – 79 that “The empirical evidence, which primarily originates from a narrow range of multinational/blue chip firms in South Africa (11), is less compelling once evidence in other sectors and countries is accounted for, and especially with respect to whether workplace programmes have the intended impact of reducing new HIV infections and HIV-related morbidity and mortality”.

Author’s response:

Thanks for this comment and for the engagement with the topic.

We have amended the title of the article to make it clear the evidence that we present is from Tanzania. We do think that our findings contribute to the broader debate and will be applicable across a range of contexts in East and West Africa.

The reviewer notes that “Institutional or workplace responses to HIV are largely dependent on national policies and strategies on HIV prevention and treatment that are in place, which are likely to differ from one country to another”. This is correct, in that there are regulations concerning employer’s responsibilities towards their workforce that will differ by country (though less so than one would imagine – for example the national policies of Tanzania, Uganda and Kenya are very similar). Often, as in the case of Tanzania, these policies primarily emphasise non-discrimination policies and rights to not be screened for HIV, although there is evidence both from the literature we review and our study that employers do not always respect these employment regulations (such as engaging in pre-employment screening for HIV which in Tanzania, Kenya, Uganda and many other countries in sub-Saharan African is not legally permitted). We have for clarity acknowledged that these national policies do play a role in the introduction.

However, the thrust of this article, which we have tried to make clear from the title onwards, is that employers are encouraged to intervene and provide comprehensive workplace programmes (similar to the ‘gold standard’ examples from South Africa) through the notion that there is a business case for doing so i.e. it is cost effective. This is an approach to encouraging employers to ‘do the right thing’ because it makes business sense rather than requiring them to do so through enforced state regulations. This approach is being applied across the sub-Saharan context, most recently highlighted in the ILO Multi-country study on workplace interventions in Africa, which is why our study can contribute to this broader debate. Proponents of the business case approach like it precisely because it seems transferable to other contexts and does not require state legislation or subsequent enforcement.

Second, there is consensus in the literature that the response from employers in the African context has been limited (drawing on work from a range of different countries which we have cited), which is consistent with our observation that there are few formal workplace programmes in Tanzania and our statement about the fact that much of the evidence regarding workplace programmes comes from a very specific setting.

We acknowledge that future research would benefit from more cross-country work and have added this to conclusion.

As a side note to the reviewer, we do think that there is a need to understand how national policies, regulatory regimes and state-employer relations shape the extent of adherence to national policies and engagement with HIV, but this is a different study and out of scope here (though we think this is what the reviewer is getting at and agree that it is important).

2. It is not evident as to why Tanzania in general and Mwanza city in particular was considered as a representative case study among the several countries in SSA and cities in Tanzania, respectively. This should be clarified.

In line with the editors and reviewers comments, we have made this clearer and there is an expanded section in the methods for the study site.

3. It is also not evident when the data collection took place and for how many days.

Author’s response: data collection took place in February and March 2017 and we have indicated that on page 7.

4. Line 102: The authors conducted 23 semi-structured interviews. For clarity, were there pre-formulated set of interview guide questions? If yes, what informed the design of the questions? In which language (English or Kiswahili) were the interviews conducted?

Author’s response: following feedback from the editors, we have significantly revised the methods section. We provide details of the interview guide and the language they were conducted in (as well as other details that have been requested by all reviewers), and have included the English and Swahili versions of the interview guide as supplementary files in our resubmission.

5. Lines 139 – 143: For clarity, consider revising the sentence “This approach (see for a full discussion of the strengths and limitations of this approach see (30) reflected the potentially sensitive topic under discussion, and in light of the linguistic limitations of one of the authors (Author A), ensured that interviews could flow without the continuous interruptions necessitated if this had been done using the research assistant or other authors as interpreters”.

Author’s response: this passage has been deleted given the need to expand the methods section

6. Line 145: State the ethical clearance certificate number.

Author’s response: ethical clearance number added. Please find this on page 8.

7. Lines 147 – 148: Which authority of local government and community issued the permission to conduct the study considering the fact that the study was not conducted at the communities? Please clarify.

Author’s response: we have added these details. Following standard ethical procedures, we gained written approval from the Mwanza Regional Medical Officer, the Regional Administrative Officer, Mwanza, and the District Medical Officers of Ilemala and Nyamagana districts (the districts in which the fieldwork was conducted). This has been added to the methods section

8. Lines 148 – 149: “…governmental and community leaders and all participants gave oral and written consent prior to the interview” Why was it necessary that all participants give oral and written consents?

Author’s response: In line with the culture in the study setting and also as recommended (please see Gordon 2000), it was important that participants consent orally as well as in writing to ensure that they are totally understand and agreed to participate in the study.

E[lisa J. Gordon](https://journals.sagepub.com/action/doSearch?target=default&ContribAuthorStored=Gordon%2C+Elisa+J), When Oral Consent Will Do. 2000, [Volume: 12 issue: 3,](https://journals.sagepub.com/toc/fmx/12/3) page(s): 235-238, [https://doi.org/10.1177/1525822X0001200304](https://doi.org/10.1177%2F1525822X0001200304)

9. Lines 148 – 149: What was the content of the written consent form?

Author’s response: we have added details in the methods section on the content of the consent form and the project information sheet

10. Lines 151 - 187: It would be more informative if the authors could first present in a tabular form, characteristics of the respondents in terms of (among other attributes) education, sex, age, years in the current position, and marital status.

Author’s response: We have provided detailed information about the characteristics of the organisations the participants are based in in Table one. However, following a request from the PLOS Global Health Editorial Office, we have removed all ages and edited the roles to ensure the anonymity of our participants. We have included the mean age of the participants.

11. Lines 152 – 154: The authors stated that “The majority of employers reported that HIV was not generally an issue for them or a relatively minor one. Some viewed it as more of a personal issue and not necessarily something that employers should get involved in, a position that is in contrast to the literature reviewed above” For clarity, the words ‘majority’ and ‘some’ need to be quantified. Further, for ease of reference, the authors could mention specific literature instead of stating the “literature reviewed above”.

Author’s response –Thanks for this comment. We would like to clarify, that qualitative approach is meant to provide a rich/detailed understanding of issues from a purposively selected sample of the population and are not statistically representative as would be the case with probability sampling in Quantitative studies. Hence, in the presentation of qualitative results, it is typical to present data using “verbal counting” e.g. majority, most, few, several, most (please see: Chang, Voils, Sandelowski, Hasselblad, & Crandell, 2009). The use of verbal counts such as “majority/most” means more than half of the total sample), “many” (between quarter to half the total sample)“few” (about a quarter the total sample). This is different from the use of the actual numbers of the participants for a particular view as is the case with quantitative studies. As noted by (Sandelowski, 2001) “Numbers do not provide the “power” in purposeful sampling the way they do in probability sampling”. It is therefore often less informative to count numbers of persons expressing a theme than to interpret the thematic lines themselves and to ensure that they are distinguished from each other. We have therefore adhered to qualitative way of presenting data in our reporting of results in this paper. We have made changes in reference to page 7, lines 37, 38 raised by the reviewer. We have indicated this sentence is referring to “majority”

12. Line 155 – 156: Improve the quote for clarity. For example, the first and second ‘its’ in line 155 need to be changed to ‘it’s’ Further, the word ‘issues’ need to be changed to ‘issue’.

Author’s response: edited

13. Line 163: Quantify ‘some employers’ for clarity.

Author’s response: this has been added as part of an earlier edit.

14. Line 166: Change the word ‘life’ to ‘lives’.

Author’s response: edited

15. Line 168: Quantify ‘other employers’ for clarity.

*Please find reference to our detailed response to a similar question in bullet 11. We hope that that answers this question too*
16. Lines 174 – 175: The statement that “… as wealthier men in the Tanzanian context are also often likely to be older and thus able to remember…” is misrepresentative in the sense that first, it is based on remarks from one respondent and second, the sample is not representative of all wealthier men in Tanzania. Further, it is important to be aware that income, education and occupation are three interrelated factors, but have different policy implications! Highly educated individuals are generally in the highest wealth quintiles than their less educated counterparts ceteris paribus.

Author’s response: This has been edited. We are not claiming our sample is representative of all men in the highest wealth quintiles! The original wording was not clear and we have now clarified that we are referring to the fact that the mean age of our sample was 49.47 years and thus men of this age could remember the earlier stages of the epidemic. Thanks for this observation.

17. Lines 178 – 179: Quantify ‘other employers’ for clarity

Author’s response: Please find reference to our detailed response to a similar question in bullet 11. We hope that that answers this question too.

18. Line 183: Quantify ‘other employers’ for clarity since the quote in lines 185 – 187 does not reflect many employers.

Author’s response: Please find reference to our detailed response to a similar question in bullet 11. We hope that that answers this question too.
19. Line 189: Quantify ‘many employers’ for clarity.

Author’s response: Please find reference to our detailed response to a similar question in bullet 11. We hope that that answers this question too.

20. Line 194: Add an ‘s’ to the word ‘avoid’.

Author’s response: edited. Thanks

21. Line 200 – 225: The findings presented here are rather surprising since prevention against the HIV/AIDS pandemic is one of the objectives set annually, virtually in every public institution, with budget allotted to implement the set target in the context of Tanzania. Virtually all quotes are from private sector. A comparative analysis of the situation in the private vs public workplaces would add value to the findings.

Author’s response: We have made clear, especially with respect to formal policies, differences between the private and public sector, as well as adding more quotes from the public sector. Thanks

22. Line 238 and 239: Change ‘its’ after the word ‘because’ and ‘contract,’ to ‘it’s’.

Author’s response: edited

23. Line 273: Consider revising the text “… but if it somewhere…” for clarity.

Author’s response: edited

24. Lines 280 – 288: Consider improving the grammar for clarity.

Author’s response: edited

25. Lines 314 – 317: Consider improving the grammar for clarity.

Author’s response: edited

26. Line 323: Incomplete sentence.

Author’s response: edited

27. Line 324 – 325: The statement that “… it does suggest an attempt to shift the responsibility” need to be rephrased as it it does not accurately reflect what the government of has always been advocating since the first case of HIV occurred in Tanzania. That is, voluntary counselling and testing. In this case, the response of the respondent in lines 319 and 320 is correct. That is, “…one employer responded with the view that this was something that should be provided by the state”.

Author’s response: this has been edited and clarified, thanks.

28. Lines 326 – 433: The discussion of the findings is generally weak. Consider restructuring the discussion such that each paragraph reflects particular finding of the study and support the discussion by existing literature from studies conducted in similar settings elsewhere. In most cases, the authors have discussed the findings of the study without accompanying evidence from other similar studies. This limits the validity of the discussions and hence, the findings presented.

Author’s response: the discussion has been significantly reworked, and references included/added

29. Line 333: Put a full-stop after the second bracket and a comma after the word ‘policies’.

Author’s response: edited

30. Line 343: The statement “…primarily ad-hoc interventions that often involve actors from the third sector” need to be rephrased considering the fact that there are formal and structured HIV/AIDS programmes in public settings in the context of Tanzania.

Author’s response: the distinction between public sector and private sector has been acknowledged above. But even in the public sector the employers did not report formal workplace programmes of the type promoted by the ILO and the business case approach. We have included quotes from public sector employers.

31. Line 345: Provide evidence to support the statement “… advocated for in the literature”.

Author’s response: This has been referenced two sentences previously, and also multiple references have been provided in the introduction

32. Line 350: Qualify the statement “…in comparison to Southern Africa”.

Author’s response: this has been addressed in the rewrite

33. Lines 355 – 356: The argument that “…having 5 HIV positive employees out of a 100 may not result in a high economic burden for employers of the magnitude reported in the mining sector in South Africa” is not supported by empirical evidence. Depending on the skills and experience, even one (1) employee, irrespective of the number of other remaining, can paralyses or cause significant disruptions of the services of an organization if the said employee is incapacitated by a health shock. I recommend that the authors reexamine the said argument.

Author’s response – in the reworking of the discussion this passage has been deleted

34. Line 376: Better use ‘in this study’ instead of ‘above’ in the statement “The evidence presented above”.

Author’s response: amended with reviewer’s suggested text

35. Line 423 – 425: The policy implication presented is largely not supported by findings from the study. It did not come out explicitly from the findings of the study that what is happening in the study areas is due to limited information or limited distribution of condoms or these would potentially reduce or lessen the negative attitudes towards PLWHIV. If information campaigns are necessary, it would be more informative to provide examples (informed by the findings of the present study) of key message to consider during the implementation of the information campaigns in order to achieve the intended purposes. Further, in order for the proposed intervention of distribution of self-testing kits to be meaningful, it would be informative if the authors could provide evidence of the contexts where such an intervention has been found to be useful in relation to the findings of the present study.

Author’s response: we have qualified this statement, and also provided references to the provision of self-testing kits in the workplace (which is being promoted by WHO/ILO).

36. Line 431: Could the authors cite examples of a local and a national NGO for clarity?

Authors’ response: an example of a INGO mentioned by our participants has been included. We have also added to this point.

37. Lines 432 – 433: There is need to provide evidence to support the argument that engagement of NGOs than employers is more likely to “safeguard the confidentiality of employees who have concerns about HIV, including regarding their serostatus”.

Author’s response – this has been expanded to reference external providers more generally

38. Line 439: Change the word ‘has’ after the word ‘that’ to ‘have’ to reflect that the statement is referring to plural (programmes and HIV policies). Further, it would be more informative to cite the referred “literature” (“…reported in the literature”) at the end of the word ‘literature’.

Author’s response- this sentence has been deleted in the rewrite of the discussion.

39. Lines 442 – 444: It is inappropriate to generalize that the workplace is not an “appropriate site of intervention due to inevitable inequities in employer-employee power relations that can increase the vulnerability of HIV-positive workers, as well as the unintended consequences of compulsory testing programmes” since in the context of Tanzania, the public service is guided by the “Standing Orders for the Public Service” that provides what public servants should do at the workplace in all work-related undertakings. It is therefore, not expected that employer-employee power relations would influence decisions in human capital development including HIV/AIDS intervention programmes that are guided by national policies on HIV/AIDS.

Author’s response – we raise this as a question, not as a definitive statement as the reviewer seems to be suggesting. We have clarified the distinction between public and private sector policies. We also suggest which HIV related policies are more suitable for the workplace, and link the issue of employer-employee relations to the more general literature on the effectiveness of workplace programmes. It is important to acknowledge that power relations will influence the success of workplace interventions. References to continued HIV stigma/discrimination in the workplace in Tanzania have been provided. Thanks.

40. Line 448: Change the word ‘of’ after the word ‘Institute’ to ‘for’ to read ‘National Institute for Medical Research’ instead of ‘National Institute of Medical Research’.

Author’s response: edited

Reviewer #2: The manuscript is well written and presents relevant subject. The presented data were collected in 2017; however, the results are still valid in the sense that they highlight the potential role of the employers when it comes to HIV interventions.

Thanks for the encouraging remarks.

Specific comments:
Include a reference of MRCC ethical approval. See lines 145-146

Author’s response: MRCC ethical clearance number added.

Indicate the qualitative analysis framework used. Eg. Content or Thematic analysis by who? Also describe who analyzed the data.

Author’s response: We have now added details on how analysis was done and indicated the analysis approach taken. Please find this on page 7.

The presentation of the findings/results should be guided by the chosen analytical approach. For example, as a reader, I would like to know whether the subtitles are 'Themes' or 'Categories'.
NB: The authors may use Critical Appraisal Skills Program (CASP) Tool to review the methods, and results sections. This will increase clarity to the readers.

Author’s response: this has been addressed with a significant overhaul of the methods section. Thanks.
