# Supplementary material for: A win-win scenario? Employers’ responses to HIV in Tanzania: A qualitative study
Source: PLOS Glob Public Health. 2022 Nov 21;2(11):e0000058. doi: 10.1371/journal.pgph.0000058 (PMC10021273; doi:10.1371/journal.pgph.0000058)
Supplement: Attachment — Submitted filename: Response to Reviewers rev2.docx [file pgph.0000058.s005.docx]

**Response to Reviewer and Journal Comments**

Journal Requirements:

1. Your co-authors, Joyce Wamoyi ([j.wamoyi@gmail.com](mailto:j.wamoyi@gmail.com)), Samwel Mgunga ([smgunga2@gmail.com](mailto:smgunga2@gmail.com)), and John Changalucha ([jchangalucha@yahoo.com](mailto:jchangalucha@yahoo.com)), have not confirmed authorship of the manuscript. We have resent them the authorship confirmation email; however please check that the above email address for them is correct and follow up personally to ensure they confirm. Please note that we cannot pass your manuscript to Production until we have received confirmations from all co-authors.

**Author Response: Author’s have not received the confirmation emails. Please resend and I will chase them up.**

2. Please update your Competing Interests statement. If you have no competing interests to declare, please state: “The authors have declared that no competing interests exist.”

**Author Response: This has been updated in Editorial Manager**

3. Please review your reference list to ensure that it is complete and correct. If you have cited papers that have been retracted, please include the rationale for doing so in the manuscript text, or remove these references and replace them with relevant current references. Any changes to the reference list should be mentioned in the rebuttal letter that accompanies your revised manuscript. If you need to cite a retracted article, indicate the article’s retracted status in the References list and also include a citation and full reference for the retraction notice.

**Author Response: All references have been reviewed. No papers have been retracted. All references have been added using reference management software.**

[Note: HTML markup is below. Please do not edit.]

Reviewers' comments:

Reviewer's Responses to Questions

**Comments to the Author**

1. If the authors have adequately addressed your comments raised in a previous round of review and you feel that this manuscript is now acceptable for publication, you may indicate that here to bypass the “Comments to the Author” section, enter your conflict of interest statement in the “Confidential to Editor” section, and submit your "Accept" recommendation.

Reviewer #1: (No Response)

2. Does this manuscript meet PLOS Global Public Health’s [publication criteria](https://journals.plos.org/globalpublichealth/s/criteria-for-publication)? Is the manuscript technically sound, and do the data support the conclusions? The manuscript must describe methodologically and ethically rigorous research with conclusions that are appropriately drawn based on the data presented.

Reviewer #1: Yes

3. Has the statistical analysis been performed appropriately and rigorously?

Reviewer #1: Yes

4. Have the authors made all data underlying the findings in their manuscript fully available (please refer to the Data Availability Statement at the start of the manuscript PDF file)?

The [PLOS Data policy](https://journals.plos.org/globalpublichealth/s/data-availability) requires authors to make all data underlying the findings described in their manuscript fully available without restriction, with rare exception. The data should be provided as part of the manuscript or its supporting information, or deposited to a public repository. For example, in addition to summary statistics, the data points behind means, medians and variance measures should be available. If there are restrictions on publicly sharing data—e.g. participant privacy or use of data from a third party—those must be specified.

Reviewer #1: No

**Author’s response: We have submitted a data statement and explanation for this to the editorial office**

5. Is the manuscript presented in an intelligible fashion and written in standard English?

PLOS Global Public Health does not copyedit accepted manuscripts, so the language in submitted articles must be clear, correct, and unambiguous. Any typographical or grammatical errors should be corrected at revision, so please note any specific errors here.

Reviewer #1: Yes

6. Review Comments to the Author

Please use the space provided to explain your answers to the questions above. You may also include additional comments for the author, including concerns about dual publication, research ethics, or publication ethics. (Please upload your review as an attachment if it exceeds 20,000 characters)

Reviewer #1: (No Response)

7. PLOS authors have the option to publish the peer review history of their article ([what does this mean?](https://journals.plos.org/globalpublichealth/s/editorial-and-peer-review-process#loc-peer-review-history)). If published, this will include your full peer review and any attached files.


**Do you want your identity to be public for this peer review?** If you choose “no”, your identity will remain anonymous but your review may still be made public.


For information about this choice, including consent withdrawal, please see our [Privacy Policy](https://www.plos.org/privacy-policy).

Reviewer #1: No

**General Comment**: The authors are have made significant improvements to the earlier version of the manuscript. However, there are some minor issues which need to be addressed for clarity and in order to improve further the manuscript before publication as detailed hereunder. In this regards, the manuscript is accepted **SUBJECT** to incorporation of the outlined comments and suggestions.

1. Line 112: add ‘is’ between ‘it’ and ‘a’

**Authors response: edited**

2. Lines 112 – 114: add a reference to support the facts presented.

**Author’s response: three references to the most recent Population-Based HIV Impact Assessments for each country have been included**

3. Line 139: delete the word ‘below’ after the word ‘Table 1’.

**Author’s response: deleted**

4. Line 144: Table captions are normally inserted above the table. Verify that the current position of the caption is consistent with the requirement of the Journal - PLOS Global Public Health

**Author’s response: Thanks for noticing this. PLOS guidelines state that Table title to be placed above the Table, so this has been edited**

5. Line 184: “…*Participants provided oral and written informed consent prior to the interview*” Why was it necessary that all participants give both oral and written consents?

**Authors response: we provided an explanation for this in our response to the first round of reviews. We do not think that this is contentious. Our participants were being asked to provide sensitive information, and in the Tanzanian context are a sample not often included in public health research. Providing written consent fulfils local institutional requirements for the procedural aspect of consent, and so this was done. However, we also believe that providing verbal consent provokes a more substantive engagement with the consent process, the content of the consent form, and the project information sheet. This can only strengthen ethical standards related to informed consent.**

6. Line 406: Provide evidence to support the statement “… *advocated for in the literature…*”.

**Author’s response: We have added a reference. This statement refers to the general literature we reviewed in the introduction.**

7. Lines 486 – 489: There is need to provide evidence to support the argument that “*Where services are provided, local and national NGOs or external medical providers may be more appropriate organising agents than employers, and will have more ability to safeguard the confidentiality of employees who have concerns about HIV, including regarding their serostatus*”.

**Author’s response: Our text makes this clear that this statement is a policy suggestion based on our findings. Employers consistently acknowledged that they understood their employees concerns around confidentiality, and also that when testing programmes were enforced employees did not turn up for them. We think this statement is consistent with these concerns. However, we have included two references from the ILO which make this clear – local/national community providers should be used.**
