# Supplementary material for: A win-win scenario? Employers’ responses to HIV in Tanzania: A qualitative study
Source: PLOS Glob Public Health. 2022 Nov 21;2(11):e0000058. doi: 10.1371/journal.pgph.0000058 (PMC10021273; doi:10.1371/journal.pgph.0000058)
Supplement: Attachment — Submitted filename: Response to Reviewers REV3.docx [file pgph.0000058.s006.docx]

The journal requests that qualitative articles include an appropriate reporting guideline, such as COREQ or SRQR, to ensure inclusion of essential elements such as clear research aims, reproducible analytic methods, and consideration of bias and limitations. Please complete these guidelines and update the manuscript accordingly - note further comments below on the key elements required to strengthen the manuscript for further consideration.

- Please include CORE-Q or SRQR (<http://journals.plos.org/globalpublichealth/s/submission-guidelines#loc-qualitative-research>) checklist and note areas for additional detail to adhere to these guidelines, such as inclusion/exclusion criteria, methods of approaching potential respondents, and reasons for non-participation. Please spell out the ‘core research questions and aims’ (line 168/169) that guided the codes earlier in the manuscript, and consider using the start of the results section to describe the overall coding framework arrived at during data analysis. The discussion section must include consideration of potential bias and limitation in the study findings, and could be streamlined to consolidate interpretation of the findings and ensure they are put in context of other research and other settings.

*Authors response:*

*We have completed the COREQ checklist and included this a supplementary file. We have added details in the methods section as per the checklist requirements where possible.*

*In terms of the specific issues raised by the reviewer, we have added/expanded the following:*

- *Inclusion/exclusion criteria*
- *Reasons for non-participation*
- *Methods for approaching participants*
- *The core research questions in lines 168/169*
- *A consideration of bias and limitations in the discussion section*

*We think that our findings are put into context with other research and other settings. The project arose from a critical assessment of the existing literature, and our article is in close dialogue with this throughout. To make this clear we have added a few more references in the discussion section. We extended the discussion section following comments from the previous reviewers, and we think that the discussion is coherent with our key findings and the data presented in the Results section*.

- The Study Setting paragraph suggests Mwanza is a relatively low HIV prevalence area, although 7% general population is not necessarily low in an absolute sense but primarily in comparison to heavily affected cities/business in South Africa where prior research has focused. It would be helpful to contextualize this in terms of the HIV epidemic and in terms of other diseases.

*Authors’ response: We have added some contextual data that illustrates progress towards the 95-95-95 targets which itself emphasises the need for the expansion of HIV testing*

- Finding around required testing for food service workers seems quite important — are employers misinterpreting or violating policy in requiring such testing? The statement, “However, it became clear that the HIV testing component of the regular health checks that food workers have to undergo is not compulsory due to regulations but is included in company contracts” is insufficient in presenting where this information comes from and whether in fact the checks are legal but not required vs. illegal, given that they can be considered employment discrimination against those testing positive. Please spell out further the interviewee’s apparent understanding of the law/policy in the Results and expand how it pertains to actual law in the discussion (or results if the correct application of the law was identified in the course of other interviews). From an ethical perspective, I wonder if the research team considered it necessary to report on this requirement if in fact it is not in keeping with current law

*Authors response*

*Thanks for this comment. This is an issue that the research team have discussed at length! When we conducted the interviews, both food producers said that testing was required due to government regulations (both quoting TFDA regulations). At a later date, we cross-checked this with the relevant government agencies, but due to a delay in analysing the data (primarily due to the lead author changing jobs), by the time we checked this, there had been institutional reforms in Tanzania, with the TFDA no longer in existence and its duties split across two different agencies. We have spoken to officials who have said that compulsory HIV tests are not in current regulations for the food sector and this has not changed recently (hence our original interpretation), but we have been unable to access written policy documents to confirm this. On further reflection, we suspect this is a mis-understanding of the regulations on the part of the employers, and we have edited this, added this to the limitations section, and also an additional policy recommendation suggesting that this regulation is clarified with food producing firms in Tanzania. Given the employers were reporting that they were operating within the regulations, at the time of the research we did not suspect that this may not be in keeping with current law, so did not consider reporting them. We also do not feel that we have strong enough evidence to do so, but hope that by highlighting this issue, relevant firms can be reminded that HIV testing is not included in routine health screening of workers in the food production sector.*

- Minor: Define 95/95/95 goals, spell out ILO on first use

*Authors’ response: Edited*
